# Brief moderate-intensity cycling, momentary nervousness, and mathematics performance in young adults

**DOI:** 10.3389/fpsyg.2026.1824734

**Published:** 2026-09-10

**Authors:** Kim Ouwehand, Myrto Mavilidi, Christine Van Nooijen, Oliver Lindemann, Mathilde de Koster, Mirko Schmidt, Liye Zou, Qian Yu, Bjorn B. De Koning, Fred Paas

**Affiliations:** 1 Department of Psychology, Education and Child Studies, Erasmus University Rotterdam, Rotterdam, Netherlands; 2 Faculty of Education, Southern Cross University, Sydney, NSW, Australia; 3 Institute of Sport Science, University Bern, Bern, Switzerland; 4 Body–Brain–Mind Laboratory, School of Sports Science and Physical Education, South China Normal University, Guangzhou, China; 5 Department of Psychology, The Ohio State University, Columbus, OH, United States; 6 South Hampton Education School, University of Southampton, Southampton, United Kingdom; 7 School of Computer Science and Engineering, University of New South Wales, Sydney, NSW, Australia

**Keywords:** cognitive load, mathematics anxiety, mathematics performance, physical exercise, task complexity, test anxiety

## Abstract

**Introduction:**

Mathematics anxiety and test anxiety are associated with poorer mathematics performance. This study investigated whether a brief bout of moderate-intensity physical activity reduces momentary nervousness and improves subsequent mathematics performance. Sixty young adults (18–29 years) were randomly assigned to either a 10-min cycling condition or a passive sedentary control condition.

**Methods:**

Momentary nervousness was assessed before the intervention, immediately afterward, and after completion of a 32-item mathematics test containing lower and higher-complexity arithmetic problems. Mathematics performance was measured using accuracy and reaction time.

**Results:**

Heart rate data confirmed successful implementation of the exercise manipulation. A mixed repeated-measures ANCOVA revealed no significant main effects of condition or time on momentary nervousness, although a Time × Condition interaction was observed. Follow-up analyses indicated that participants in the cycling condition reported higher nervousness at baseline than participants in the control condition, suggesting that regression to the mean may have contributed to the observed pattern. For mathematics performance, a significant Condition × Complexity interaction emerged for accuracy, *F*(1, 54) = 5.79, *p* = 0.020, ηp^2^ = 0.10, indicating that the relation between condition and performance differed across problem complexity levels.

**Discussion:**

However, because no significant main effects of condition were found for either accuracy or reaction time, the accuracy interaction should be interpreted with caution with regard to the overall mathematics performance. Larger studies using validated state-anxiety measures, active control conditions, and more precise performance measures are needed to clarify potential boundary conditions of acute exercise effects.

## Introduction

Rapid technological change has increased the demand for a workforce with strong competencies in Science, Technology, Engineering and Mathematics (STEM). However, performances in STEM-related school subjects’ courses, particularly mathematics, appear to be declining (Barroso, 2020; Nilsen et al., 2022). Poor performance in mathematics does not only originate from cognitive deficits (Caviola et al., 2022). Many children and adults report experiencing strong negative emotional reactions toward mathematics in general, which can severely disrupt their learning and performance (Ashcraft, 2002; Živković et al., 2023). This phenomenon, commonly termed mathematics anxiety, is associated with lower mathematics achievement and broader academic underperformance. It is also linked to the avoidance of mathematical activities (Daker et al., 2021) and reduced engagement with STEM coursework, which may in turn shape later educational trajectories and career choices (Demedts et al., 2022). This study investigated whether a short physical activity intervention can reduce anxiety and improve performance on a mathematics test.

Mathematics anxiety is commonly defined as “a feeling of tension and anxiety that interferes with the manipulation of numbers and the solving of mathematical problems in ordinary life and academic situations” (Richardson and Suinn, 1972, p.551). Although it is associated with test anxiety and broader forms of anxiety, it is not simply a domain-general manifestation of either construct. Moreover, mathematics anxiety is conceptually and empirically distinct from mathematical learning disorders, such as developmental dyscalculia (for recent review see, e.g., Cipora et al., 2022). Across educational levels, mathematics anxiety shows robust negative associations with mathematical achievement, with detrimental effects observed across a broad range of mathematical-related tasks (Dowker et al., 2016; Yarkwah et al., 2024).

In contrast to mathematics anxiety, mathematics self-concept refers to an individual’s perception and evaluation of their own competence in mathematics (Marsh and O'Neill, 1984). Students with a stronger mathematics self-concept generally demonstrate higher mathematics achievement and greater confidence when engaging in mathematical tasks. Furthermore, mathematics self-concept is closely related to both achievement and emotional experiences in mathematics. Research has shown that students with a more positive mathematics self-concept tend to report lower levels of mathematics anxiety and better mathematics performance, whereas negative self-perceptions are associated with poorer outcomes (Justicia-Galiano et al., 2017; Van der Beek et al., 2017). Accordingly, mathematics self-concept may influence how individuals approach and perform mathematics tasks and was therefore included in the present study as an important covariate when examining the associations among exercise, nervousness, and mathematics performance.

Several studies observed negative correlations between mathematics anxiety and math achievement (see Dowker (2021) for recent review). Mathematics anxiety can impair performance by consuming working memory resources needed for problem-solving and information retrieval. This mechanism can be framed within Cognitive Load Theory (CLT), which distinguishes between intrinsic and extraneous cognitive load. Intrinsic load is driven by task complexity, often operationalized as element interactivity, that is, the degree to which multiple information elements must be simultaneously maintained and integrated in working memory (Sweller, 2010). Importantly, mathematics anxiety particularly impairs performance in complex arithmetic tasks (so-called anxiety-complexity effect; Ashcraft and Faust, 1994). That is, tasks with multi-step arithmetic require coordinating intermediate results and procedures, thereby increasing intrinsic load and the working memory demands of the task (e.g., Chen et al., 2023). Complexity can further increase with larger operands and smaller numerical distance, which can elevate processing demands and element interactivity (Ashcraft and Faust, 1994). In contrast, extraneous load reflects demands that are not essential to the task itself, such as suboptimal information presentation or internal stressors. Mathematics anxiety may therefore be conceptualized as an internal source of extraneous load: anxiety-related worry and physiological arousal compete for working memory resources, disproportionately disrupting performance when intrinsic load is already high. Conversely, perceived competence and confidence in one’s mathematical ability are associated with better performance, potentially by reducing maladaptive, resource-consuming thoughts (Lee and Kung, 2018). Recent integrative accounts further suggest that bodily states and sensorimotor engagement can modulate cognitive demands and learning-related processing, providing a framework for interpreting when physical activity may support versus hinder performance under varying task complexity (Castro-Alonso et al., 2024; Skulmowski, 2024; Zou et al., 2025).

However, the precise cognitive mechanisms underlying the negative correlation between anxiety and performance remain controversial. While many researchers state that impaired mathematics performance is the result of mathematics anxiety by consuming cognitive resources (see above), other researchers argue that poor math performance and deficits in basic number processing may also contribute to the development of mathematics anxiety (e.g., Maloney and Beilock, 2012). In other words, mathematics anxiety may partially reflect previous experiences of difficulty in mathematics. Students with lower mathematical competence may experience greater anxiety during performance situations, whereas practice and increased competence may reduce both anxiety and performance-related concerns. Therefore, mathematical competence and anxiety should be interpreted as related, but distinct, constructs.

A substantial body of research indicates that physical activity or exercise can reduce stress (Mücke et al., 2018) and attenuate stress-related physiological responses (Caplin et al., 2021; Hamer et al., 2006). Physical activity has also been linked to higher enjoyment and better academic outcomes (e.g., Egger et al., 2019). Reviews of acute physical activity, defined as the transient effects of a single bout, report small-to-moderate benefits for cognition across the lifespan (Chang et al., 2012; Mavilidi et al., 2025; Moreau and Chou, 2019; Pontifex et al., 2019).

Recent theorizing further proposes that effects of physical activity on achievement may be mediated by both domain-general and domain-specific executive functions, a distinction that is particularly relevant for mathematics tasks that vary in complexity (Kalén et al., 2021; Zhang et al., 2026). These reviews primarily examined cognitive outcomes (e.g., executive functions, attention, and memory) rather than mathematics performance per se. In contrast, more consistent evidence for improvements in adolescents’ mathematics performance comes from studies on chronic physical activity exposure (Álvarez-Bueno et al., 2017; Sneck et al., 2019; Watson et al., 2017).

Pontifex et al. (2019) concluded that evidence for acute exercise effects remains inconclusive for specific types of executive functions (e.g., working memory and cognitive flexibility), as well as for memory and intelligence or achievement outcomes. Consistent with this mixed picture, Hsieh et al. (2025) reported that a 20-min bout of aerobic exercise (treadmill walking/jogging) improved young adults’ performance on inhibitory control tasks, yet these behavioral gains were not paralleled by changes in the neurophysiological measures assessed (Hsieh et al., 2025). Given the well-established association between working memory and mathematics performance (Raghubar et al., 2010), exercise-related improvements in working memory could plausibly translate into better mathematics outcomes. However, the existing evidence is insufficient to support strong causal claims, and further work is needed to specify boundary conditions. Recent classroom research similarly suggests that brief pre-class physical exercise may produce selective benefits for mathematics-relevant inhibitory control rather than broad improvements across executive functions, consistent with the view that acute exercise effects depend on domain and task-alignment (Zhang et al., 2026). Related arguments propose that exercise-cognition literature should more explicitly address when and why effects may be absent or context dependent, which is particularly relevant for studies looking at acute-bouts (Herold et al., 2025). One influential account attributes the acute exercise effect to arousal modulation, often described by an inverted-U or J-shaped relation in which moderate arousal optimizes performance and very low or high arousal is less beneficial (McMorris and Hale, 2015).

Regarding exercise intensity, a meta-analytical review including participants from children to older adults reported small positive effects of acute high intensity exercise on executive functioning relative to resting control conditions (Moreau and Chou, 2019). Notably, effects did not differ meaningfully between high and moderate-intensity exercise. Experimental findings in young adults are similarly mixed. Jung et al. (2023) tested whether 15 min of acute moderate-intensity treadmill walking enhances mathematical creativity and reported no performance differences in undergraduate students (Jung et al., 2023). In contrast, Hsieh et al. (2025) observed improved inhibitory control following moderate-intensity treadmill walking. One plausible explanation for these discrepant findings is the fidelity of intensity manipulation: Hsieh et al. monitored and regulated heart rate to approximately 70% of age-predicted maximal heart rate, whereas Jung et al. (2023) did not report the extent to which the walking bout increased heart rate. Consequently, differences in achieved physiological intensity may have contributed to the divergent cognitive outcomes.

A recent umbrella review underscores that the effects of physical activity on mental health and well-being are context dependent (Vella et al., 2023). Specifically, characteristics such as setting (indoor vs. outdoors), social format (individual vs. group-based), and exercise modality (aerobic vs. resistance training) may differentially predict which outcomes improve and to what extent. In adult populations, multiple syntheses support the effectiveness of physical activity for reducing symptoms of depression and anxiety (McDowell et al., 2019; Pavey et al., 2011; Rebar et al., 2015; Salihu et al., 2021; Singh et al., 2023). Evidence also suggests that exercise intensity can matter for stress-related physiological responding: in young adults, higher-intensity exercise tends to yield larger reductions in stress-related blood pressure, with bouts of at least ~30 min showing benefits when aerobic demand reaches approximately 50% of VO_₂max_ (Hamer et al., 2006). Despite this broader literature linking physical activity with anxiety-related outcomes, studies that examine physical activity, specifically in relation to mathematics anxiety, appear to be lacking.

Only limited work has examined whether brief exercise bouts performed immediately before mathematics assessment influence performance and anxiety. For example, Mavilidi et al. (2020) investigated short bouts of exercise prior to a mathematics test in children. More recently, a cluster-randomized classroom trial reported that brief pre-class exercise improved mathematics-specific inhibitory control measured after mathematics instruction, suggesting a plausible mechanism through which acute exercise bouts could support mathematics-related performance (Zhang et al., 2026). However, neither found reliable intervention effects on mathematics performance or anxiety. A key limitation noted by the authors was insufficient control of exercise intensity (i.e., the extent to which children achieved the intended activity level), which may have attenuated or obscured effects.

Another factor that might have affected the results is the general physical fitness of the participants. We suggest that participants in good physical condition due to regular exercise might respond differently to physical activity intervention than those who are less fit, as hypothesized (Schmidt et al., 2021) and shown in previous studies investigating the moderating role of individual characteristics such as aerobic fitness (Jäger et al., 2015) or habitual physical activity (Zhu et al., 2023). Therefore, we included a subjective physical fitness self-rating to be able to control for general fitness.

Building on these findings, the present study argues that individual difference variables should be explicitly modeled when testing exercise effects on mathematics outcomes. In particular, mathematics anxiety, test anxiety, mathematics self-concept, and physical fitness are well-established predictors of mathematics performance and may moderate responsiveness to acute exercise. Accounting for these learner characteristics can help isolate the specific contribution of exercise and clarify the conditions under which it is beneficial. To our knowledge, few acute-exercise studies in young adults have examined mathematics performance while experimentally manipulating task complexity and concurrently assessing both mathematics anxiety and test anxiety.

The present intervention study examined whether a brief 10-min bout of heart-rate–controlled, moderate-intensity cycling reduces momentary nervousness and improves subsequent mathematics test performance relative to a passive sedentary control condition. We formulated five hypotheses. First, mathematics anxiety, test anxiety, and momentary nervousness were expected to be positively correlated (H1). Second, mathematics anxiety and test anxiety were expected to be negatively associated with mathematics accuracy and positively associated with reaction time (H2). Third, mathematics self-concept and self-rated physical fitness were expected to be positively associated with accuracy and negatively associated with reaction time (H3). Fourth, momentary nervousness was expected to decrease following the intervention in the exercise group, evidenced by a larger reduction from baseline to post-intervention and end-of-experiment ratings relative to the passive sedentary control group (H4). Fifth, the exercise group was expected to show higher subsequent mathematics performance than the control group, with the largest benefits for complex problems (H5).

## Method

### Participants

Participants were 60 undergraduate Psychology students (9 men, 49 women, 2 undisclosed; *M*_age_ = 20.03 years, *SD* = 2.52 years, range 18–29 years) who registered on a website of the hosting university for enrollments in psychological research. *A priori* power analysis conducted in G*power 3.1 assumed a large effect of *f* = 0.40, *α* = 0.05, power of 0.80, two groups and four covariates, yielding a minimum required sample of 52 participants. As the final analyses involved repeated measures and more covariates than assumed in this calculation, the study was adequately powered only to detect relatively large effects. Small or moderate condition and interaction effects may therefore have remained undetected. The advertisement on this website specified that students with heart conditions or those who use heart medication, such as beta-blockers, could not participate. Prior to the study, approval was obtained from the Human Ethics Research Committee of the Erasmus University Rotterdam (file number: ETH2425-0465). All participants were randomly assigned to either the exercise group (*n* = 30) or the passive sedentary control group (*n* = 30).

## Materials

### Part one: pre-existing trait measures

Participants filled out a survey consisting of eligibility confirmation (i.e., having no heart issues), a declaration of consent to collect and use participants’ anonymized data for research and educational purposes, demographics (age, academic year, gender), mathematics anxiety scale (Suinn and Winston, 2003), test anxiety scale (Driscoll, 2007), mathematics self-concept scale Marsh and O'Neill (1984), and the international fitness scale (Ortega et al., 2011). Lab assessments included measures of momentary nervousness, mathematics performance, and heart rate (which will be described more in detail later).

*Mathematics anxiety* was measured with a shortened version of the mathematics anxiety Rating Scale by Suinn and Winston (2003), consisting of 30 items presenting hypothetical scenarios related to mathematics. Participants had to estimate the level of expected anxiety in the hypothetical scenario on a five-point Likert scale ranging from “1—Not at all anxious” to “5—Extremely anxious.” An example scenario is: “Taking an examination (final) in a mathematics course.”

*Test anxiety* was measured with the Westside Test Anxiety Scale by Driscoll (2007) asking participants to rate 10 test situations on a five-point Likert scale ranging from “1—Not at all true” to “5—Extremely true or Always true.” Participants were asked to rate how true each statement for them was. An example item is: “The closer I am to a major exam, the harder it is for me to concentrate on the material.”

*Self-rated physical fitness* was measured using the International Fitness Scale by Ortega et al. (2011) consisting of five items on a five-point Likert scale ranging from “1—poor” to “5—very good.” Each item asked participants to “Please try to think about your level of physical fitness (compared to your friends) and choose the right option.” An example item is: “Your general physical fitness is: (participants could choose to fill the blanks by selecting one response from very poor to very good, see Appendix B).”

*Mathematical self-concept* was measured using a 10-item subscale of the Self-Description Questionnaire-III (SDQ) by Marsh and O'Neill (1984) designed to measure participants’ perceptions on their own mathematical abilities. Participants could rate their perceptions from “1—Completely disagree” to “5—Completely agree.” An example item is: “I find many mathematical problems interesting and challenging.”

For all pre-existing trait measures, mean scores (sum score/number of items) were used for analysis. All complete questionnaires are provided in Appendix A.

### Part two: dependent variables

*Momentary nervousness* was assessed as a proxy of state anxiety at three time points using the single item, “How nervous are you at this moment?,” rated from “1—very low” to “9—very high.” Since the item assesses one component of momentary nervousness rather than the full multidimensional construct, it is described as a single-item state-anxiety rating.

The mathematics test was developed by the authors and consisted of 32 arithmetic verification problems across four categories: addition, subtraction, fractions, and division. Each category comprised four lower-complexity and four higher-complexity items. Problems were presented together with a proposed solution (e.g., *2 + 2 = 4*), and participants were required to indicate whether the statement was correct or incorrect. All test items are provided in Appendix B.

Problem complexity was based on the work of Ashcraft and Faust (1994). Lower-complexity items involved relatively small operands and fewer computational demands, whereas higher-complexity items involved larger operands and more demanding calculations.

Performance was assessed using the proportion of correct responses (accuracy) and mean reaction time (RT) for correct responses only. Accuracy and reaction times were calculated separately for lower and higher-complexity problems. Participants scoring below chance level (< 0.50 accuracy) were excluded from the analyses; however, no participants met this criterion. Accuracy scores ranged from 0.69 to 0.88 for lower-complexity problems and from 0.56 to 1.00 for higher-complexity problems.

Raw reaction times for correct responses were screened prior to analysis. A lower bound of 500 ms was specified to identify implausibly fast responses. Since the observed reaction times ranged from 724 ms to 20,214 ms, no trials were excluded based on this criterion. Error rates were generally low. In the exercise condition, error rates were 6.04 and 5.63% for lower and higher-complexity problems, respectively. In the passive sedentary control condition, error rates were 8.30 and 16.04%, respectively.

Inspection of the reaction-time distributions indicated positive skewness across all condition × complexity cells, which is common for RT data. Therefore, natural log-transformed reaction times were used in the primary analyses. Inspection of the transformed distributions indicated substantially improved normality (see Supplementary materials for details). Mean raw reaction times ranged from 1902 to 5,893 ms for lower-complexity problems and from 3,200 to 9,641 ms for higher-complexity problems.

### Hardware

**Figure 1:**
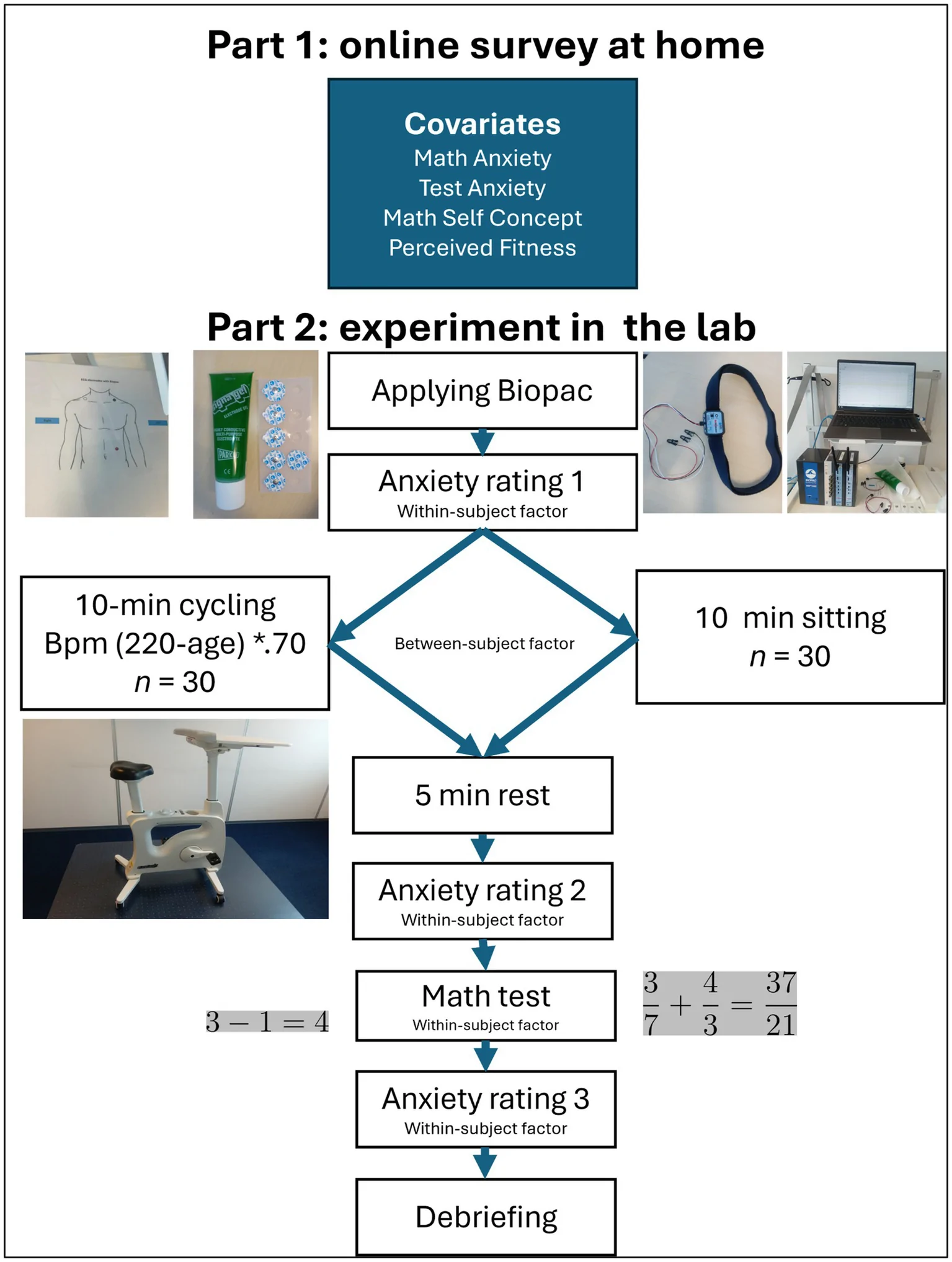
Schematic representation of the experimental procedure. Participants completed an online survey, followed by a laboratory session including a baseline assessment of momentary nervousness (MN1), an exercise or passive sedentary manipulation, a second assessment of momentary nervousness (MN2), a mathematics test, and a final assessment of momentary nervousness (MN3).

The heart rate (in beats per minute, BPM) was measured using the BIOPAC MP160 system in combination with the Dual Wireless Electrocardiogram BioNomadix Transmitter, Parker Signagel® Electrode Gel, BIOPAC General Purpose Disposable EL503 Electrodes, and BioNomadix Electrode Leads (3′45 cm). HP-desktop PCs were used to present momentary nervousness and mathematics performance test. For the exercise intervention, a deskbike was used (see Figure 1).

### Software

Qualtrics Qualtrics (2024) was used to administer all pre-measures. E-Prime 3.0 (Psychology Software Tools, Inc., 2016) was used to present the momentary nervousness ratings and the mathematics performance task. The BIOPAC system used AcqKnowledge (version 4) to record heart rate.

## Procedure

The study consisted of two parts. In the first part, pre-measures for mathematics anxiety, test anxiety, mathematics self-concept, and physical fitness were taken at home using an online questionnaire in Qualtrics. The second part took place in the lab, where participants were enrolled in a 10-min exercise or passive sedentary control (10 min sitting while waiting) condition, after which they completed a mathematics test.

### Part one

After signing up, participants received an email that included a link to an online Qualtrics survey, which took 15–20 min to complete. Participants who completed the survey received a three-digit number at the end and were instructed to use it for scheduling a lab appointment.

### Part two

Upon arrival at the laboratory, participants were screened again for cardiovascular conditions and the use of heart-rate–altering medication. After eligibility had been reconfirmed, participants received a standardized explanation of the heart-rate measurement procedure and were connected to the BIOPAC recording system. Participants then provided a baseline rating of momentary nervousness (MN1).

Randomization to condition (exercise vs. passive sedentary control; 1:1 allocation ratio) was implemented using a computer-generated random sequence created by the primary researcher before data collection commenced. No blocking or stratification procedures were applied. Participants were enrolled by the experimenter and assigned sequentially according to the pre-generated allocation list in the order in which they attended their laboratory appointment. Condition assignment was revealed to the experimenter immediately prior to the intervention. As the experimenter had access to the allocation list, allocation concealment was not implemented. However, laboratory appointments were scheduled independently of condition assignment, reducing the likelihood that participants could be selectively enrolled into a specific condition.

Participants were subsequently assigned to either the exercise condition (10 min of moderate-intensity cycling) or the passive sedentary control condition, in which they remained seated quietly for 10 min and performed no additional task. Momentary nervousness was assessed immediately after the intervention (MN2) and again at the end of the experiment following completion of the mathematics test (MN3). The lack of allocation concealment should be considered when interpreting the findings because it may have introduced a risk of selection or performance bias.

For the heart rate recording, the electrodes were positioned above the right collarbone (white), above the left collarbone (black), and below the left rib cage (red).

To determine the target heart rate for moderate-intensity exercise, the guidelines of the American College of Sports Medicine were applied (Liguori, 2020). The calculation followed the formula: (220—age) × 0.70. The first step, 220 minus age, provided the estimated maximum heart rate in BPM. Multiplying this value by 0.70 yielded 70% of the maximum heart rate, corresponding to a moderate-intensity workload. A tolerance range of ±10 BPM was used.

Heart rates were monitored for all participants, but for those in the cycling condition, participants were instructed to keep their heart rate within their personal a target range ± 10 bpm. If it fell outside the target range, the experiment leader encouraged participants to cycle faster or slower. After 10 min, there was a 5-min rest period, followed by the second momentary nervousness rating. Participants then completed the mathematics test. Finally, the last momentary nervousness rating was completed, and participants were debriefed about the experiment. The duration of the experiment was approximately 40 min.

## Planned data processing and statistical analyses

### Preliminary analyses

As a manipulation check, we conducted a one-way ANOVA to compare mean heart rate during the intervention between conditional groups. Next, a one-way MANOVA was conducted to examine whether participants in the cycling and control conditions differed on the covariate’s mathematics anxiety, test anxiety, fitness, and mathematics self-concept.

Correlational analyses were done to explore relations between the pre-exiting trait measures (test anxiety, mathematics anxiety, self-rated physical fitness, and mathematics self-concept) and the outcome measures (momentary nervousness, mathematics accuracy and mathematics reaction times).

### Main analyses

For the analysis of momentary nervousness, a 2 × 3 repeated-measures ANCOVA was conducted with a between-subject factor for condition (exercise vs. passive sedentary) and a within subject factor for time (MN1 measured at baseline, MN2 measures after the intervention and MN3 at the end of the experiment) and covariates (mathematics anxiety, test anxiety, self-rated fitness, and mathematics self-concept).

Performance accuracy and reaction times (on log-transformed reaction times) were analyzed by two 2 × 2 mixed ANCOVAs with a between-subject factor for condition (exercise vs. passive sedentary) and a within-subject factor for problem complexity (simple vs. complex) and covariates (mathematics anxiety, test anxiety, fitness, and mathematics self-concept).

Partial eta-squared (η_p_^2^) was calculated as a measure of effect size for *F-*values, with values of 0.01, 0.06, and 0.14, characterizing small, medium, and large effect sizes, respectively (Cohen, 1988). Cohen’s *d* was calculated as a measure of effect size for *t-*values, with values of 0.20, 0.50, and 0.80, characterizing small, medium, and large effect sizes, respectively (Cohen, 1988).

## Results

### Preliminary analyses

#### Heart rate

Heart rate measures were consistently higher in the cycling condition than in the control condition, *F*(1, 58) = 804.67, *p* < 0.001, η_p_^2^ = 0.93. *M*_BMP_) was 140.97 (*SD* = 4.32) in the cycling condition and 82.10 (*SD* = 10.51) in the control condition. *M*_BMP_ was 142.33 (*SD* = 4.17) and 80.56 (*SD* = 9.89), respectively. The lower and upper bounds of heart rate during 80% of the observation period were also higher in the cycling condition [95% CI (134.59 and 147.37)] than in the control condition (95% CI [72.87 and 91.95], respectively).

#### Pre-existing trait measurements

The multivariate effect of condition was not significant, Pillai’s Trace = 0.07, *F*(4, 55) = 0.97, *p* = 0.433, ηp^2^ = 0.07. Follow-up univariate analyses showed no significant differences between conditions for mathematics anxiety, *F*(1, 58) = 0.44, *p* = 0.511, ηp^2^ = 0.01, test anxiety, *F*(1, 58) = 1.00, *p* = 0.323, ηp^2^ = 0.02, self-rated fitness, *F*(1, 58) = 0.14, *p* = 0.707, ηp^2^ < 0.01, or mathematics self-concept, *F*(1, 58) = 0.14, *p* = 0.714, ηp^2^ < 0.01. Thus, the conditional groups did not differ significantly depending on baseline characteristics.

#### Correlations

After a Bonferonni correction for multiple comparisons with a corrected significance level of 0.05/45 = 0.001, the following correlations were significant: Mathematics anxiety was strongly associated with test anxiety, *r*(58) = 0.60, *p* < 0.001. MN1 (momentary nervousness measured at the start of the task) was strongly related to MN2 (momentary nervousness after the manipulation), *r*(58) = 0.79, *p* < 0.001, and moderately to strongly related to MN3 (momentary nervousness at the end of the experiment), *r*(58) = 0.48, *p* < 0.001. MN2 was also strongly associated with MN3 at the end of the task, *r*(58) = 0.71, *p* < 0.001. Finally, reaction times on easy and difficult problems were strongly correlated, *r*(58) = 0.60, *p* < 0.001.

#### Effect of intervention on momentary nervousness

The mixed ANCOVA, using the Greenhouse–Geisser correction revealed no significant main effect of condition, *F*(1, 54) = 1.56, *p* = 0.218, ηp^2^ = 0.03, nor time, *F*(1.73, 93.38) = 1.05, *p* = 0.344, ηp^2^ = 0.02. However, the time × test anxiety interaction was significant, *F*(1.73, 93.38) = 4.89, *p* = 0.013, ηp^2^ = 0.08, as was the time × condition interaction, *F*(1.73, 93.38) = 3.46, *p* = 0.042, ηp^2^ = 0.06. This result indicates that changes in momentary nervousness over time differed between the cycling and control conditions, although the effect size was relatively small. No interaction effects were observed between time and mathematics anxiety, *F*(1.73, 93.38) = 2.00, *p* = 0.147, ηp^2^ = 0.04, self-rated fitness, *F*(1.73, 93.38) = 0.27, *p* = 0.733, ηp^2^ = 0.01, or mathematical self-concept, *F*(1.73, 93.38) = 0.84, *p* = 0.421, ηp^2^ = 0.02.

**Figure 2:**
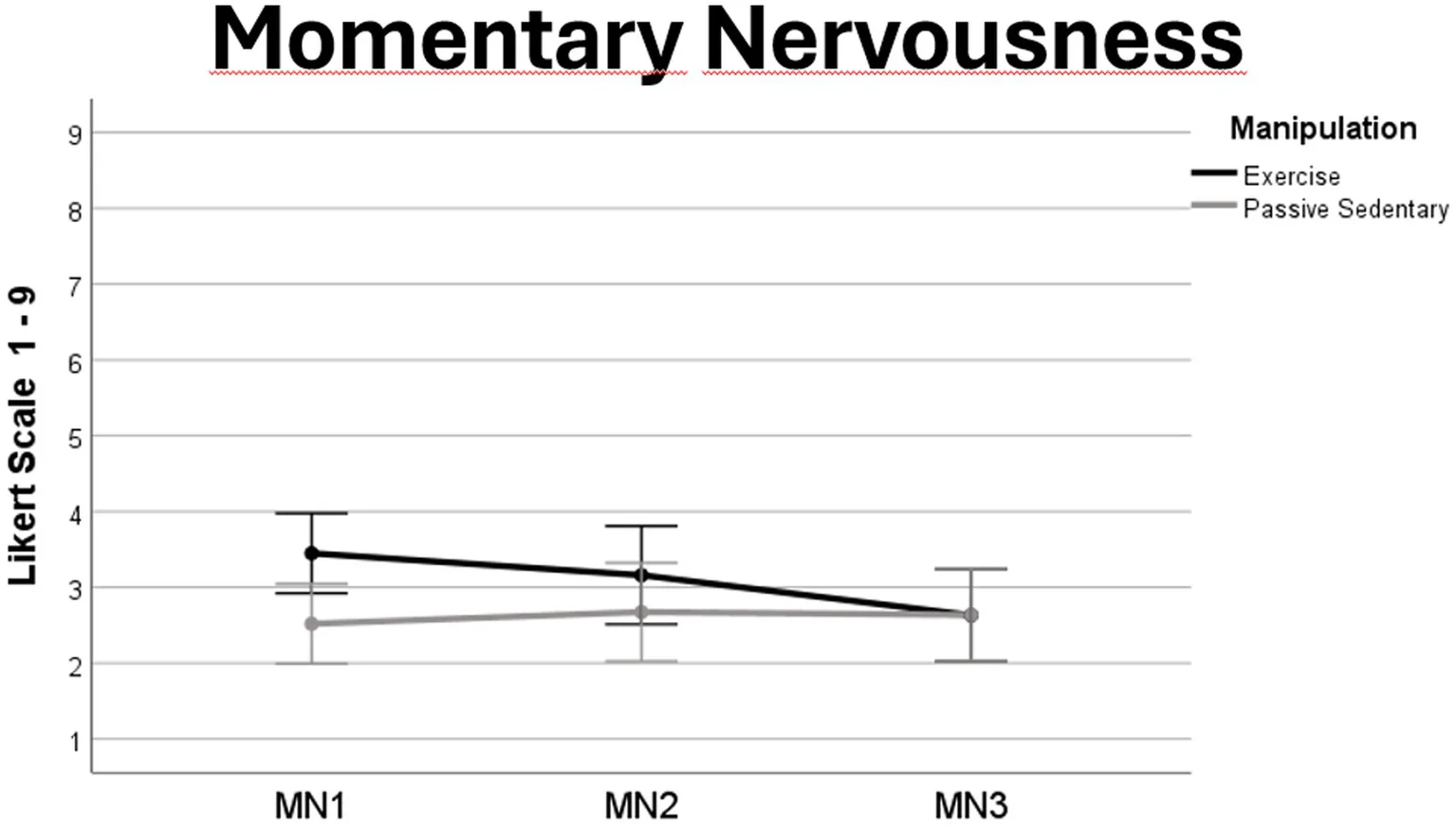
Momentary Nervousness ratings of the Exercise and Passive Sedentary groups measured before the manipulation (MN1), after the manipulation (MN2), and at the end of the experiment (MN3). Error bars represent ±2 standard errors.

Follow-up analyses were conducted to examine group differences at each measurement occasion with a Bonferroni correction adapting the significance level to 0.05/3–0.017. A significant effect of condition was found for MN1, 1, *F* (1, 58) = 6.01, *p* = 0.017, ηp^2^ = 0.10, reflecting participants in the exercise condition reporting higher momentary nervousness than participants in the control condition. No significant differences between conditions were observed for MN2, *F* (1, 58) = 1.09, *p* = 0.301, ηp^2^ = 0.02, or MN3, *F* (1, 58) < 0.01, *p* = 0.992, ηp^2^ < 0.01. These findings suggest that group differences in momentary nervousness were largest at the beginning of the experiment and decreased across time. From visual inspection of Figure 2, it seems that the exercise group started somewhat more nervous than the control group, but that nervousness levels regressed to the mean during the experiment.

## Effect of intervention on mathematics performance

### Accuracy

A 2 (condition) × 2 (problem complexity: simple vs. complex) mixed ANCOVA was conducted on accuracy scores while controlling for mathematics anxiety, test anxiety, fitness, and mathematics self-concept. There was no significant main effect of complexity, *F*(1, 54) < 0.01, *p* = 0.961, ηp^2^ < 0.01, and no significant main effect of condition, *F*(1, 54) = 1.85, *p* = 0.179, ηp^2^ = 0.03.

However, a significant condition × complexity interaction emerged, *F*(1, 54) = 5.79, *p* = 0.020, ηp^2^ = 0.10. In the exercise condition, accuracy was slightly higher for complex problems [*M* = 0.85, 95% CI (0.81, 0.90)] than for simple problems [*M* = 0.83, 95% CI (0.81, 0.85)]. In contrast, in the control condition, accuracy was lower for complex problems [*M* = 0.79, 95% CI (0.75, 0.84)] than for simple problems [*M* = 0.84, 95% CI (0.82, 0.86)], Table 1.

**Table 1:** Correlations between covariates and momentary nervousness measured at baseline (MN1), after the manipulation (MN2) and at the end of the experiment (MN3) and mathematics performance.

Correlations										
	TA	Fit	MSC	MN1	MN2	MN3	AcS	AcC	RTS	RTC
Mathematics anxiety (MA)	0.60^*^	−0.29	0.05	0.30	0.31	0.23	−0.21	−0.06	0.31	0.02
Test anxiety (TA)		−0.24	0.05	0.35	0.20	0.08	−0.12	0.17	0.30	0.18
Fitness (Fit)			0.16	−0.24	−0.15	−0.12	0.30^*^	−0.01	−0.18	0.01
Mathematics self concept (MSC)				0.02	0.11	0.18	−0.08	−0.04	−0.10	−0.10
Momentary nervousness1 (MN1)					0.79^**^	0.48^**^	−0.14	0.28	−0.01	−0.05
Momentary Nervousness1 (MN2)						0.71^**^	−0.22	0.12	−0.06	−0.07
Momentary nervousness1 (MN3)							−0.07	<0.01	−0.08	−0.15
Acc simple problems (AcS)								0.29	−0.05	0.03
Acc complex problems (AcC)									< 0.01	0.05
RT simple problems (RTS)										0.66^**^
RT complex problems (RTC)										

**Correlation is significant at the 0.001 level (2-tailed). *N* = 60.

A significant complexity × test Anxiety interaction was also found, *F*(1, 54) = 6.36, *p* = 0.015, ηp^2^ = 0.11, indicating that the relationship between test anxiety and performance differed between simple and complex problems. No interactions were observed for complexity with mathematics anxiety, *F*(1, 54) = 2.81, *p* = 0.099, ηp^2^ = 0.05, self-rated fitness, *F*(1, 54) = 1.23, *p* = 0.273, ηp^2^ = 0.02, or mathematical self-concept, *F*(1, 54) = 0.04, *p* = 0.853, ηp^2^ < 0.01.

### Reaction time

There was no significant main effect of complexity, *F*(1, 54) = 1.62, *p* = 0.208, ηp^2^ = 0.03, and no significant complexity × condition interaction, *F*(1, 54) = 0.01, *p* = 0.941, ηp^2^ < 0.01. The between-subjects main effect of condition approached significance but did not reach the conventional *α*-level, *F*(1, 54) = 3.44, *p* = 0.069, ηp^2^ = 0.06. Adjusted means were numerically lower in the exercise condition than in the passive sedentary control condition, but the corresponding confidence intervals overlapped substantially.

A significant complexity × mathematics anxiety interaction was observed, *F*(1, 54) = 5.20, *p* = 0.027, ηp^2^ = 0.09, indicating that the association between mathematics anxiety and reaction time differed as a function of problem complexity. The complexity × test anxiety interaction was not significant, *F*(1, 54) = 3.41, *p* = 0.070, ηp^2^ = 0.06. No significant interactions were found between complexity and self-rated fitness, *F*(1, 54) = 0.79, *p* = 0.379, ηp^2^ = 0.01, or mathematics self-concept, *F*(1, 54) = 0.06, *p* = 0.801, ηp^2^ < 0.01.

Estimated marginal means indicated that participants in the exercise condition showed adjusted mean log-transformed reaction times of 7.86 [*SE* = 0.05, 95% CI (7.77, 7.95)] for simple problems and 8.57 [*SE* = 0.05, 95% CI (8.47, 8.67)] for complex problems. Corresponding adjusted means in the passive sedentary control condition were 7.98 [*SE* = 0.05, 95% CI (7.89, 8.08)] for simple problems and 8.69 [*SE* = 0.05, 95% CI (8.59, 8.79)] for complex problems. For means and SD’s of the raw reaction times and unadjusted log-transformed scores, see Table 2.

**Table 2:** Descriptive statistics of the trait measures (mathematics anxiety, test anxiety, physical fitness rating, mathematics self-concept), raw and adjusted (for covariates) accuracy and RT means and standard deviations.

	Exercise			Passive sedentary		
	Raw M (SD)	LN	Adjusted^ln^ M (SE)	Raw M (SD)	LN	Adjusted^ln^ M (SE)
Mathematics anxiety*	2.62 (0.62)			2.51 (0.59)		
Test anxiety*	2.65 (0.90)			2.87 (0.84)		
Physical fitness rating*	3.31 (0.78)			3.25 (0.57)		
Mathematics self-concept*	4.76 (0.27)			4.79 (0.29)		
MN1	3.37 (1.75)			2.60 (1.25)		
MN2	3.17 (2.04)			2.67 (1.49)		
MN3	2.67 (1.90)			2.60 (1.38)		
Acc simple problems	0.83 (0.05)		0.83 (0.01)	0.84(0.05)		0.84 (0.01)
Acc complex problems	0.84 (0.12)		0.85 (0.02)	0.80(0.11)		0.79 (0.02)
RT simple problems	3,475 (938)	7.86 (0.28)	7.86 (0.05)	3,729 (887)	7.98 (0.24)	7.98 (0.05)
RT complex problems	6,210 (1616)	8.56 (0.28)	8.69 (0.05)	6,987 (1741)	8.70 (0.26)	8.69 (0.05)

*Covariate used to calculate the adjusted means.

^ln^ Natural log transformation.

## Discussion

In this study, we investigated the effect of a single bout of a 10-min exercise on subsequent momentary nervousness levels and mathematics performance on low and high complexity problems. A unique contribution of this study is that we investigated this while controlling for participants’ pre-existing levels of mathematics and test anxiety, mathematics self-concept, and self-rated physical fitness. H1 was partially supported. Mathematics anxiety correlated with test anxiety and with all three momentary nervousness ratings, whereas test anxiety did not. H2 received limited support. Mathematics anxiety moderated the relation between problem complexity and reaction time, whereas evidence for a similar moderating role of test anxiety was weaker. No relations between test anxiety and mathematics performance was found.

H3 was not supported because self-rated physical fitness and mathematics self-concept were unrelated to the performance measures. With regard to H4, the primary omnibus pattern suggested differential change across time between conditions, but baseline imbalance and possible regression-to-the-mean effects prevent a causal interpretation. H5 received partial support. A significant condition × complexity interaction emerged for accuracy, suggesting that the relationship between condition and performance differed as a function of problem complexity. However, because no overall condition effect was observed and no comparable interaction emerged for reaction time, the practical significance of this finding remains uncertain and warrants replication.

As recent classroom studies suggests selective effects on mathematics-specific inhibition following brief pre-class exercise (Zhang et al., 2026), future studies could test domain-relevant executive-function measures (e.g., mathematics-specific inhibitory control) more directly alongside anxiety and cognitive-load indicators. This interpretation is consistent with accounts that explicitly link bodily states to changes in cognitive processing demands within a cognitive-load framework (Zhang et al., 2025; Zou et al., 2025). The present findings that performance on complex problems improved for the exercise groups seems to align with this. However, this study did not include a direct measure of subjective cognitive load or perceived difficulty ratings, which would be informative for evaluating a cognitive-load account of the complexity effect. Although adjusted mean reaction times were numerically longer for complex than simple problems, variability and covariate adjustment resulted in a non-significant complexity effect.

Despite the largely null findings, the present study has several strengths. First, participants were randomly assigned to conditions, reducing the likelihood that pre-existing individual differences systematically biased the results. Second, the exercise intervention was monitored using heart-rate recordings, allowing verification that participants in the cycling condition achieved the intended moderate-intensity exercise level. Third, the study simultaneously accounted for several theoretically relevant individual-difference variables, including mathematics anxiety, test anxiety, mathematics self-concept, and self-rated physical fitness, thereby providing a more rigorous test of exercise effects than studies that do not control for these factors. Fourth, mathematics performance was assessed using both accuracy and reaction-time measures and included problems that varied in complexity, enabling examination of whether exercise effects differed across task demands. Finally, reaction-time analyses were conducted using log-transformed data to address distributional skewness, increasing the robustness of the statistical inferences. Together, these design features strengthen confidence that the observed findings reflect the specific intervention tested rather than obvious differences between participant groups or measurement artifacts.

A notable limitation is the baseline imbalance in momentary nervousness between conditions. Despite random assignment, participants in the exercise group reported higher nervousness at the start of the experiment than participants in the control group. As a result, the subsequent reduction in nervousness observed in the exercise group may partly reflect regression to the mean rather than a true intervention effect. Although the revised analyses accounted for baseline differences statistically, the possibility that initial group differences influenced the anxiety findings cannot be excluded. Consequently, conclusions regarding the effect of brief cycling on momentary nervousness should be interpreted with caution.

Another limitation of this study concerns the characteristics of the sample. Participants were predominantly female undergraduate psychology students, reflecting the composition of the recruitment pool. Consequently, the findings may not generalize to male students, students from other academic disciplines, or broader young-adult populations. Future research should examine whether similar effects emerge in more diverse and gender-balanced samples.

Furthermore, the present study used a proxy of anxiety was assessed with a single item asking about momentary nervousness. Although this reduced participant burden during repeated assessment, the item does not provide evidence about internal consistency and may not capture the cognitive, affective, and physiological dimensions of momentary nervousness. The anxiety findings should therefore be interpreted as changes in self-reported momentary nervousness rather than as definitive changes in the broader construct of momentary nervousness. Future studies should use a validated multi-item state-anxiety measure. Baseline momentary nervousness was also somewhat higher in the exercise group than in the passive sedentary group. Although this difference did not reach statistical significance, regression-to-the-mean effects cannot be excluded, and the anxiety findings should therefore be interpreted with caution.

An important methodological limitation is the absence of an active control condition. As participants in the comparison group remained seated and inactive, expectancy effects, experimenter attention, and engagement-related factors cannot be ruled out as alternative explanations for the observed reductions in momentary nervousness. Consequently, the findings should not be interpreted as definitive evidence of an exercise-specific effect. Future studies should include an active control condition matched for time, attention, and engagement.

A further limitation concerns statistical precision. The *a priori* sample-size calculation was based on a large, expected effect (*f* = 0.40), whereas the final analyses involved repeated measures and additional covariates. Consequently, the study was primarily powered to detect relatively large intervention effects, and smaller condition and condition × complexity effects may have gone undetected. This limitation is reflected in the width of several confidence intervals and in the uncertainty surrounding the estimated interaction effects. Therefore, the absence of statistically significant intervention effects should not be interpreted as evidence that smaller effects do not exist. Future studies would benefit from larger samples that allow more precise estimation of exercise-related effects on anxiety and mathematics performance.

Finally, the study did not include a direct measure of prior mathematical competence or achievement. Given that mathematics anxiety and mathematics performance are often reciprocally related, it remains unclear to what extent the observed anxiety levels reflect emotional responses, mathematical ability, or both. Future research should include standardized measures of mathematical competence to better disentangle emotional and knowledge-related influences on performance.

Another point for future research would be to investigate whether contextual factors moderate the effects of acute exercise on anxiety and mathematics performance. For example, combining brief physical activity with exposure to natural environments may influence cognitive restoration and anxiety regulation, potentially enhancing academic performance outcomes (Rhee et al., 2023; Vella et al., 2023). With technological tools, such as virtual reality, we suggest it would also be interesting to investigate whether immersiveness can boost the influence of scenery. Exploring these venues will not only add to the scientific knowledge about the relations between exercise, surroundings, anxiety and cognitive performance, but also allow for concrete recommendations to implement effective interventions in practice (Mavilidi et al., 2026).

From an educational perspective, this study’s findings do not support recommending brief pre-test cycling as a method for reducing anxiety or improving mathematics performance. Replication with larger and more diverse samples, validated multi-item anxiety measures, standardized measures of prior mathematics competence, verified exercise intensity, concealed allocation, and an active control condition are needed before practical recommendations can be made.

## Data Availability

The original contributions presented in the study are included in the article/Supplementary material, further inquiries can be directed to the corresponding author.
